# Assessment of the trophic state of a hypersaline-carbonatic environment: Vermelha Lagoon (Brazil)

**DOI:** 10.1371/journal.pone.0184819

**Published:** 2017-09-21

**Authors:** Lazaro Laut, Maria Virginia Alves Martins, Fabrizio Frontalini, João M. Ballalai, Pierre Belart, Renan Habib, Luiz F. Fontana, Iara M. M. M. Clemente, Maria Lucia Lorini, João G. Mendonça Filho, Vanessa M. Laut, Marcos de Souza Lima Figueiredo

**Affiliations:** 1 Laboratório de Micropaleontologia – LabMicro, Universidade Federal do Estado do Rio de Janeiro – UNIRIO, Rio de Janeiro, Rio de Janeiro, Brazil; 2 Departamento de Estratigrafia e Paleontologia, Universidade do Estado do Rio de Janeiro – UERJ, Rio de Janeiro, Rio de Janeiro, Brazil; 3 Unidade de Investigação GeoBioTec, Departamento de Geociências, Universidade de Aveiro, Aveiro, Portugal; 4 Dipartimento di Scienze Pure e Applicate (DiSPeA), Università degli Studi di Urbino "Carlo Bo", Urbino, Italy; 5 Laboratório de Palinofácies & Fácies Orgânicas (LAFO), Universidade Federal do Rio de Janeiro – UFRJ, Rio de Janeiro, Rio de Janeiro, Brazil; 6 Departamento de Ciências Naturais, Universidade Federal do Estado do Rio de Janeiro – UNIRIO, Rio de Janeiro, Rio de Janeiro, Brazil; 7 Programa de Pós-Graduação em Biologia Marinha e Ambientes Costeiros, Universidade Federal Fluminense – UFF, Instituto de Biologia, Niterói, Rio de Janeiro, Brazil; 8 Programa de Pós-graduação em Biodiversidade Neotropical, Universidade Federal do Estado do Rio de Janeiro – UNIRIO, Rio de Janeiro, Rio de Janeiro, Brazil; Universidade de Aveiro, PORTUGAL

## Abstract

Vermelha Lagoon is a hypersaline shallow transitional ecosystem in the state of Rio de Janeiro (Brazil). This lagoon is located in the protected area of Massambaba, between the cities of Araruama and Saquarema (Brazil), and displays two quite uncommon particularities: it exhibits carbonate sedimentation and displays the development of Holocene stromatolites. Due to both particularities, the salt industry and property speculation have been, increasingly, generating anthropic pressures on this ecosystem. This study aims to apply a multiproxy approach to evaluate the trophic state of Vermelha Lagoon based on physicochemical parameters and geochemical data for the quantification and qualification of organic matter (OM), namely total organic carbon (TOC), total sulfur (TS), total phosphorus (TP) and biopolymeric carbon (BPC), including carbohydrates (CHO), lipids (LIP) and proteins (PTN). The CHO/TOC ratio values suggest that OM supplied to the sediment is of autochthonous origin and results, essentially, from microbial activity. The cluster analyses allowed the identification of four regions in Vermelha Lagoon. The Region I included stations located in shallow areas of the eastern sector of Vermelha lagoon affected by the impact of the artificial channel of connection with Araruama Lagoon. The Region II, under the influence of salt pans, is characterized by the highest values of BPC, namely CHO promoted by microbiological activity. The Region III include stations spread through the lagoon with high values of dissolved oxygen and lower values of TP. Stromatolites and microbial mattes growth was observed in some stations of this sector. Region IV, where the highest values of TOC and TS were found, represents depocenters of organic matter, located in general in depressed areas. Results of this work evidences that the Vermelha Lagoon is an eutrophic but alkaline and well oxygenated environment (at both water column and surface sediment) where the autotrophic activity is greater than heterotrophic one. These particular conditions make this a special and rare ecosystem.

## Introduction

Hypersaline environments are distributed throughout several regions of the world associated with tropical and arid climates [1]. Hypersaline coastal lagoons are commonly shallow environments strongly influenced by wind action that promotes water column homogenization [2]. The shallowness and the reduced water column further enhance the role of the bottom sediments in these ecosystems [3].

The east coast of the state of Rio de Janeiro is characterized by the presence of a large number of lagoons [4]. The occurrence of Northeast trade winds at Cabo Frio promotes the development of upwelling zone in Rio de Janeiro Coast (between Saquarema and Búzios). A climatic setting with semi-arid characteristics within a tropical environment is established in this region [5]. The hydrographic basin of Araruama lagoon is surrounded by several hypersaline lagoons, such as Pitanguinha, Pernambuco, Azul and Vermelha [6].

These hypersaline lagoons in the state of Rio de Janeiro have been studied in recent decades, since they are rare biogeochemical systems [7], [8], [9], [10]. They have been considered analogue environments to the shallow-water continental shelves of the early Earth characterized by the presence of microbial mats and underlying sediments recognized in ancient strata. The study of the whole system allows for the identification of the combination of processes that induce carbonate precipitation in bottom sediments, both in the present and in the past [10].

The abundance and composition of organic materials in coastal sediments depend on a complex combination of factors, which involves sources and physicochemical processes occurring in overlying water layers and in the sediment itself [11]. The use of the organic sediment biochemical composition can be considered a useful and sensitive tool for classifying the trophic state of marine and coastal ecosystems [12]. Several ratios among biopolymers have been proven useful proxies for assessing the origin of sedimentary organic matter and have been widely used in marine, riverine and some lagoonal environments in temperate and Mediterranean regions [11], [12], [13], [14], [15], [16], [17]. These methodologies were recently used to evaluate the trophic state of sediments in Brazilian aquatic intertropical ecosystems [18], [19] and have significantly contributed to the identification of the most polluted regions, as well as for assessing the effects of organic matter on the microbiota [11], [20], [21]. However, these methods have not yet been applied in semi-arid lagoons with carbonate sedimentation.

Carbonate environments are predominantly found in warmer tropical or subtropical oceanic regions, where carbonate-secreting organisms bloom [1]. The subtidal sediment of Vermelha Lagoon consists of a poorly-sorted mixture of skeletal sand (mollusks, ostracods and foraminifers), silica skeletal silt (diatoms frustules) and terrigenous mud and sand [22]. Thus, the distribution of carbonate content and sediment grain size in this lagoon depends more on the type and distribution of the organisms that grow in the area, than on the transport of siliciclastic sediments carried out by wind or runoff. Hence, changes in the hydrological budget, caused either by climatic change or human activity, have the potential to alter the lagoon depth and water chemistry. These changes, in turn, may affect the physiological responses of the organisms and the biotic composition of the lagoon [23].

In this context, this study aims to use a multiproxy approach to evaluate the trophic state and environmental quality of a hypersaline and carbonate coastal system, Vermelha Lagoon, in Rio de Janeiro State (Brazil). This approach is based on the evaluation of total organic carbon (TOC), total sulfur (TS), total phosphorus (TP) and biopolymer (BPC) content and physicochemical water parameters.

## Materials and methods

### Study area

**Fig 1:**
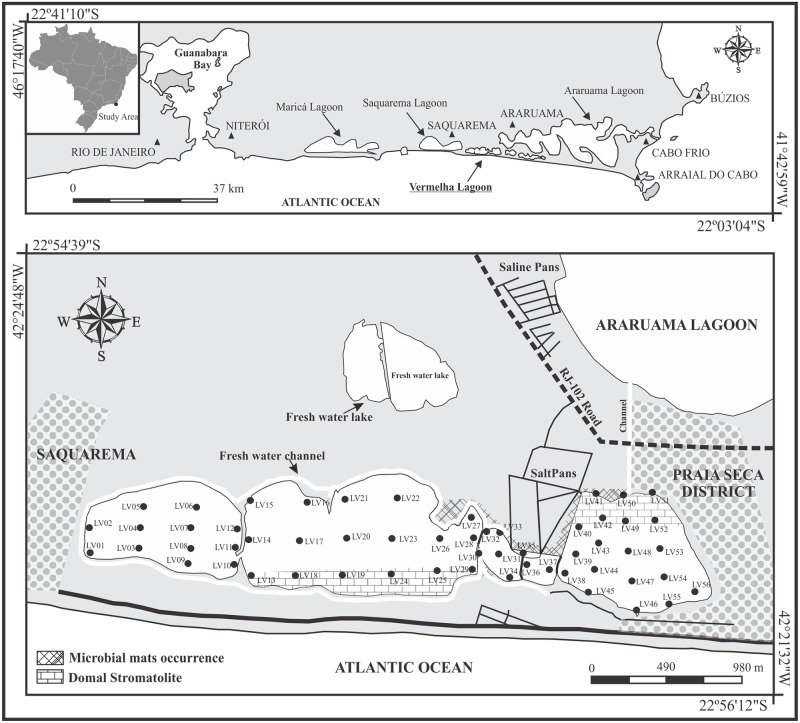
Study area in Vermelha Lagoon. The studied stations are labeled with numbers. The main towns, stromatolitic, microbial mats and salt pans are also indicated.

Vermelha Lagoon is situated between the cities of Araruama and Saquarema (22°55’S, 42°25’W) in the Massambaba Environmental Protection Area, at approximately 110 km from the city of Rio de Janeiro (Fig 1). It is 4.3 km in length, has a 10.88 km perimeter, 0.75 km maximum width and covers an area of ca. 2.5 km^2^. The water depth in this lagoon has on average 2 m [10].

The geomorphology of the region where Vermelha Lagoon is located is dominated by hills and lowlands. The main elevations are the mountains of Sapiatiba, Sapiatiba Mirim and Palmital band and the hills of Cabo Frio, such as Miranda, Forno, Atalaia, Cabo and Farol [24] The local geology is composed by Quaternary sandbar deposits and recent dune fields (aeolian deposits) [25]. Radiocarbon analyses of shells from different sediment cores recovered from Vermelha Lagoon provided an age between 3,800 and 4,200 years BP, corresponding probably to the beginning of its formation [26]. The region presents relatively low rainfall (854 mm/year), high annual evaporation rates (between 1,200 and 1,400 mm), average temperature of 23°C, insolation between 200 and 220h/month, summer with the predominance of NE winds and winter marked by discontinuous S-SW winds periods [27]. The climatic peculiarities of the Cabo Frio region have been explained by factors such as the great distance from the coast line to the Serra do Mar and the presence of an intermittent coastal resurgence, intensified by strong NE winds (Barbieri, 1984). Although considered a local and intermittent phenomenon and with low amplitude [28]), the coastal resurgence in Cabo Frio determines a peculiar semi-arid and warm microclimate, with low rainfall and high annual evaporation rates (Barbieri, 1984) in this region when compared with the rest of the southeastern Brazilian coast [27]. The climate is classified as equatorial savanna (Aw—precipitation less than 60 mm) in accordance to Kottek et al. [29] with annual temperatures around 25°C. Precipitation reaches the highest values between November and March.

The vegetation cover of this region reflects this great geomorphological diversity, in addition to the paleoevolutionary history of the Brazilian southeast and the current climate. It is reminiscent of vegetation existing during the drier and colder Pleistocene glacial periods [24]. It comprises mainly resting formations, Atlantic forest and low-trees that cover the coastal region between Arraial do Cabo and Armação de Búzios, as well as mangroves and flooded environments (swamps and lagoon banks). It has a rich flora with unique phytogeographic links and several endemic species [30]. Recently the native forests have been almost completely removed and replaced by pastures in the lowlands and hills. Small isolated patches of forest in the mountains of Palmital and Sapiatiba still remain [30].

In 1981, a fresh water channel about 3 m deep was constructed around the lagoon (Fig 1) to prevent sub-superficial water inflow [26]. Thus, nowadays Vermelha Lagoon is not receiving freshwater directly from rivers and streams. Also in 1981, the lagoon was artificially divided into three compartments for the construction of salt pans for salt extraction [31]. According to [6], this ecosystem is connected to the Araruama Lagoon through a ditch inside the salt pan area. In addition, during the field work it was observed an artificial channel also connecting both lagoons (Fig 1). Thus, the water balance of Vermelha Lagoon is controlled by rainfall, evaporation and the inflow of fresh groundwater and waters received trough the Araruama Lagoon [26].

Vermelha Lagoon is the most hypersaline coastal lagoon in Brazil (recording salinities of about 60 on average; maximum 120) and its name is related to the huge proliferation of microbial mats and the purplish bacteria that give a reddish appearance to bottom sediment [9]. Structures of stromatolites with three development stages composed of overlapping layers of sediment, precipitated minerals and coccoid and filamentous cyanobacteria are present in Vermelha Lagoon [32]. The domal structure of stromatolites can be observed in the margins of the eastern and central sectors of the lagoon (Fig 1).

According to Mansur et al. [33], Vermelha Lagoon has a very important potential use for educational, scientific and geotouristic activities due to its particular geomorphology and sedimentology (including mineralogy). Its current protection status is regulated by the Massambaba Environmental Protection Area, where it is inserted. The lagoon represents a fragile ecosystem that is threatened by climatic changes and anthropogenic influences.

### Sample methods

Fifty-six stations (LV1 to LV56) located within Vermelha Lagoon were sampled in January 2013 (Fig 1; S1 Table). Each sampled station was georeferenced with a GPS (model GPSMAP^®^ 78S—10 meters of accuracy). Physicochemical data such as salinity, temperature, dissolved oxygen and pH were obtained in the sediment superficial layer (0–1 cm) with a multiparameter probe (SANXIN SX751—Accuracy: pH: ±0.01 and oxygen: 0.01 mg/L). The sediment samples were collected by an Ekman grab aboard a kayak. The first upper centimeter of sediment was recovered and used in this study.

The sediment samples collected in each station for total organic carbon (TOC), total sulfur (TS), total phosphorous (TP) and biopolymer content analysis were placed in referenced plastic bags and preserved in cold conditions. Once in the laboratory, the sediment samples were homogenized and lyophilized in a Liotop lyophilizer (48 h) before the analysis [34].

### Total organic carbon and total sulfur

The <75 μm fractions in triplicate of each sample were homogenized, lyophilized and grounded in porcelain crucibles. Aliquots of ca. 0.26 g were weighed (0.1 mg precision) in porous, previously weighed, crucibles. In order to eliminate the carbonate fraction, HCl volumes (1:1 v/v) sufficient to cover the sample were added to the crucibles for 24 h. Before the acid reaction, the sediment residues were rinsed with distilled water until complete elimination of HCl (to pH ~ 6). Sample residues were dried at 65°C for 3 h and weighed in order to calculate the percentage of insoluble residue. TOC and TS measurements were performed with a carbon and sulphur analyzer (LECO SC 144) according to the procedure described by Mendonça-Filho et al. [34] and following the ASTM D 4239 [35]. The TOC/TS ratio, commonly referred to as C/S, was determined for each station.

### Total phosphorus

The sediment samples were dried at 50°C, homogenized and ground. Phosphorus analyses were performed by molecular absorption spectrophotometry with the following procedure. Inorganic phosphorus (IP) was measured by 1 M HCl extraction (24 h, room temperature); total phosphorus (TP) was measured by 1 M HCl extraction after ignition of the sediment (24 h, 550 (C) and; organic phosporus (OP) was determined by the difference between TP and IP [36] [37]. The TP analysis was done in triplicate.

### Biopolymer concentrations

Protein (PTN) content determinations were carried out after extractions with NaOH (0.5 M, 4 h) and were determined according to Hartree [38], modified by Rice [39] to compensate for phenol interference. Concentrations are reported as albumin equivalents. Carbohydrates (CHO) were analyzed according to Gerchacov and Hachter [40] and expressed as glucose equivalents. The method is based on the same principle as the widely used method of Dubois et al. [41], but specifically adapted for CHO determination in sediments. Lipids (LIP) were extracted by direct elution with chloroform and methanol and analyzed according to Marsh and Weinstein [42]. Lipid concentrations are reported as tripalmitine equivalents. For each biochemical analysis, blanks were prepared with the same sediment samples as previously treated in a muffle furnace (450°C, 24 h). All analyses were carried out with 3–5 replicates. Protein, carbohydrate and lipid concentrations were converted to carbon equivalents by using the following conversion factors: 0.49, 0.40 and 0.75 μg C g^-1^, respectively. The sum of PTN, CHO and LIP carbon was referred to as biopolymeric carbon (BPC) [43]. The PTN/CHO and CHO/TOC ratios were used to discriminate differences in the organic matter composition.

### Interpolation maps

Spatial interpolation is a method used to estimate data in contiguous areas and forecast the values at unknown locations, i.e., where no measured values are available, using available observation data [44]. The goal of spatial interpolation is to create a surface that is intended to best represent the empirical reality [45]. Interpolation methods can be classified in two major groups: deterministic and geostatistical. Deterministic interpolation techniques create surfaces from measured points, based on either the extent of similarity or the degree of smoothing [46]. Geostatistical or stochastic interpolation techniques capitalize on the spatial correlation between neighboring observations to predict attributed values at unsampled locations [47]. These methods are not simply based on an estimation of the unknown value as a function of distance, since they also implement the function of unknown spatial autocorrelation between the sample point values [46]. In this study, we used the ArcGIS 10.3 software to create surfaces applying four interpolation methods: three deterministic (IDW, Spline, Spline with Barriers) and one geostatistical (Kriging).

Model parameter settings and interpolation model tests are quite important. We performed sensitivity analysis varying setting parameters and model choices for the three interpolation methods to select the values that produced the best fitting model and better resulting surfaces. For all interpolation techniques, the number of nearest input sample points to be used to perform the interpolation were set as 4, 6, 8, 10 and 12. For IDW, we tested the power exponent of distance as 1, 2 and 3. For Spline we tested the regularized and tension type. Additionally, because the spline method disregards geographical barriers, we also tested the Spline with Barriers method, that treats the current grid-based surface model as an elastic membrane to achieve an approximation to a minimum curvature surface that considers both the input point data and discontinuities encoded in the barriers [48]. For Kriging, we tested the Spherical, Exponential and Gaussian semivariogram models. The best combination of interpolation techniques and parameters was selected using 2D and 3D visualization analysis to compare the generated surfaces, screening for surfaces with good behavior and avoiding anomalies and unwanted patterns (e.g., sharp edges, “bulls-eyes”). Considering the dataset, the best surfaces produced for Vermelha Lagoon were those generated by the Spline with Barriers interpolation method.

### Statistical analysis

Q-mode Cluster Analysis based on the Euclidian Distance and Ward Linkage was applied aiming to order the stations in groups with similar characteristics in terms of quantity and quality of organic matter, physical and chemical parameters. All data were normalized with the square root of 0.5 before the statistical analysis in PCord 5.0 software.

## Results

**Fig 2:**
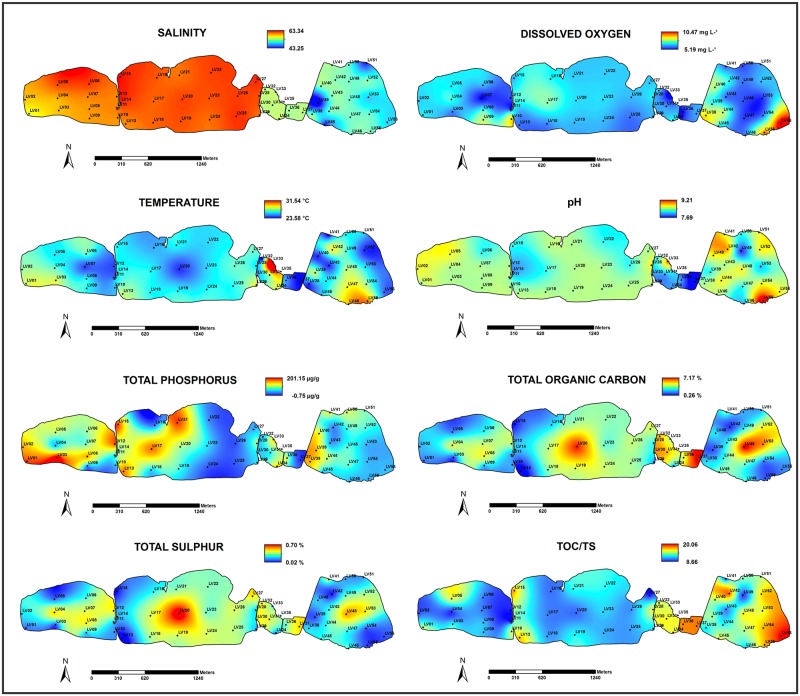
Distribution maps of salinity, dissolved oxygen (mg L^-1^), temperature (°C), pH, total sulphur (%), total organic carbon (%), total phosphorus (μg g^-1^) and TOC/S ratio values.

**Fig 3:**
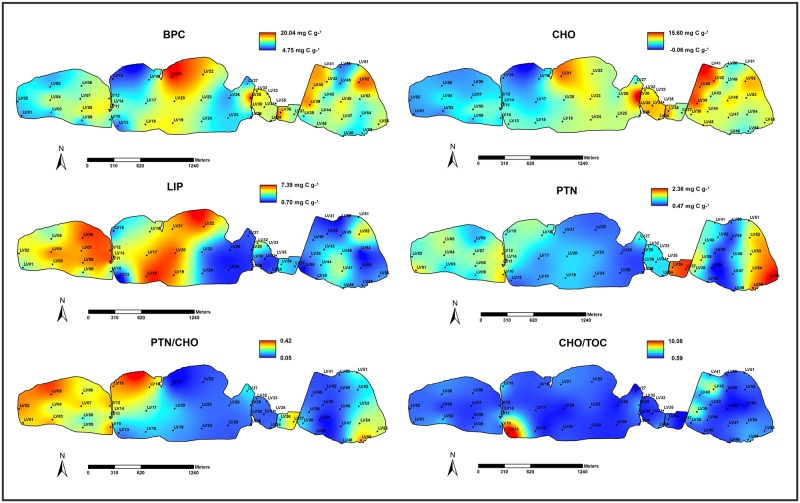
Distribution maps of biopolymeric carbon (BPC; mg C g^-1^), carbohydrate (CHO; mg C g^-1^), lipid (LIP; mg C g^-1^) and protein (PTN; mg C g^-1^) concentrations, and PTN/CHO and CHO/TOC ratios values.

Distribution maps for salinity, dissolved oxygen (mg L^-1^), temperature (°C), pH, TS (%), TOC (%), TP (μg g^-1^) and C/S ratio values are presented in Fig 2. Distribution maps for BPC, CHO, LIP, PTN, the ratios PTN/CHO and CHO/TOC were included in Fig 3.

### Physicochemical parameters

The salinity of Vermelha Lagoon varied from 43.37 in LV 39 to 63.1 in LV15, with a mean value of 56.8 during the sampling period. The values were homogeneous and higher in the western and central sectors of the lagoon, whereas the lowest salinities were recorded in the eastern sector (Fig 2, S1 Table). Dissolved oxygen varied from 5.20 mg L^-1^ to 9.2 mg L^-1^ at stations LV33 and LV55, respectively (mean 6.98 mg L^-1^). Highest dissolved oxygen contents were mainly found in the southern marginal areas of the eastern and western sectors and next to the salt pans (Fig 2, S1 Table). Temperature ranged from 23.6°C to 31.5°C at stations LV41 and LV33, respectively (mean 25.3°C). The highest values were mainly recorded in the marginal areas of the eastern and western sectors and next to salt pans (Fig 2, S1 Table). The lowest values were found mainly in the central zones of each lagoonal compartment and at north of the eastern sector close to the residential area (Fig 2). The mean pH was 8.1 and ranged from 7.7 and 9.2 at stations LV37 and LV30, respectively. The lowest pH values were found, in general, next to the salt pans (Fig 2, S1 Table).

### Organic matter and biopolymers concentrations

TS content ranged from 0.02% to 0.7% (0.3 ± 0.13%), with higher values in the central part of the lagoon and markedly low values in the peripheral margins of the western and eastern lagoonal parts. TOC content varied from 0.28% to 7.1% (3.40% ± 1.55). Higher TOC values were found in the central and eastern parts of the lagoon (Fig 2, S1 Table).

TP concentrations ranged from 16.07 μg g^-1^ to 182.08 μg/g in stations LV37 and LV03, respectively (mean 78.46 ± 40.89 μg g^-1^). The highest TP contents were recorded in the western side of each lagoonal compartment and near the central area, close to the salt pans (Fig 2, S1 Table). The TOC/S ratio values ranged from 8.96 to 20 at stations LV11 and LV30, respectively (Fig 2, S1 Table). The highest values of this ratio were found in the eastern lagoonal sector and in areas close to the salt pans (Fig 2, S1 Table).

BPC concentrations ranged from 15.01 to 43.36 mg C g^-1^ (mean 24.36 ± 6.81 mg C g^-1^). The highest BPC concentrations were recorded in the margin where microbial mats are found, in the central sector and near the salt pan area (Fig 3, S1 Table). Similar distribution patterns were observed for CHO (6.26–38.38 mg C g^-1^; mean 16.81 ± 7.12 mg C g^-1^) and PTN (1.13–4.79 mg C g^-1^; mean 2.49 ± 0.9 mg C g^-1^), but not for LIP (1.86–9.72 mg C g^-1^; mean 4.9 ± 2.48 mg C g^-1^) (Fig 3). LIP concentrations are in general low through the Vermelha Lagoon. The highest values of LIP were recorded in the western and central sectors (Fig 3, S1 Table).

The PTN/CHO ratio (ranging from 0.06–0.35) was quite uniform along the lagoon, where most of the stations presented a mean value of 0.17 (Fig 3; S1 Table). The main exceptions were observed in the western sector, where PTN/CHO values reached 0.35 and at station LV56 and in the extremities of the eastern lagoonal sector, where the lowest value (0.25) was recorded (Fig 3, S1 Table). CHO/TOC values varied between 0.71–10 (Fig 3; S1 Table). CHO/TOC values increased significantly at stations LV10 and LV13, located in the margin of the central sector of the lagoon.

### Statistical analyses

**Fig 4:**
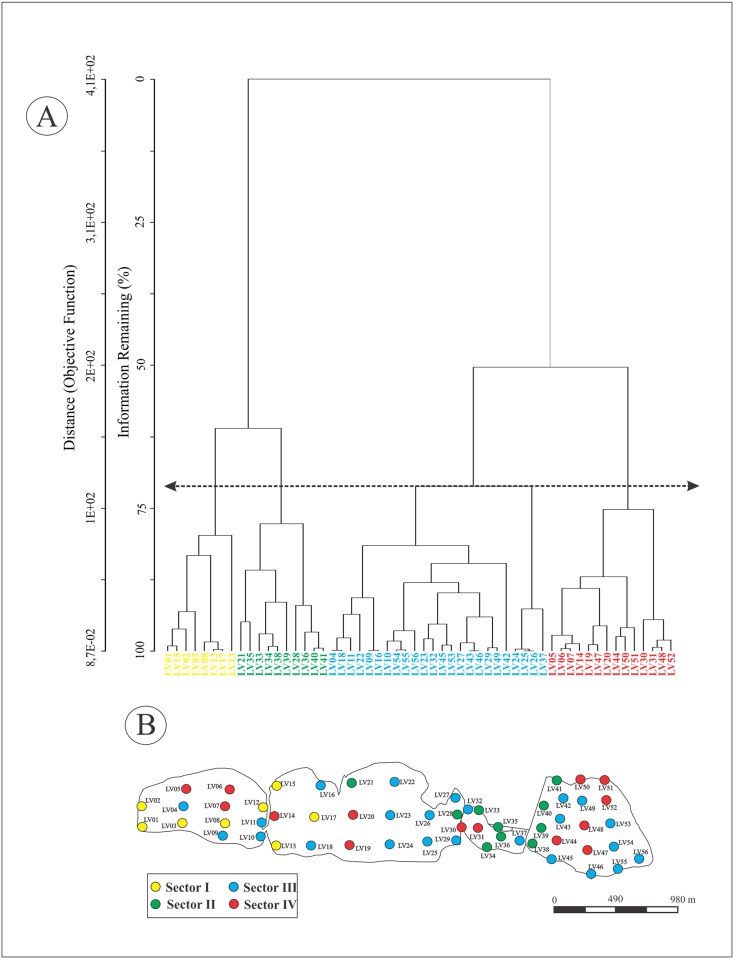
The cluster analysis results based on the physicochemical parameters and geochemical data. A) Cluster dendrogram plot relating the studied stations. B) Distribution map of the station groups identified through the cluster analysis at Vermelha Lagoon.

Based on cluster analysis results with 70% of similarity, four station groups were identified (Fig 4A). Each sector can be easily identified on the Vermelha Lagoon map (Fig 4B). According to these results: Group I—includes stations located mostly in the western (LV01, LV02, LV03, LV08 and LV12) and central (LV13, LV15 and LV17) sectors of the lagoon; Group II is composed mostly by stations located near salt pain margin (such as LV28, LV33, LV34, LV35, LV36, LV38, LV39, LV40 and LV41); Group III contains the largest number of stations with distribution in all sectors of the lagoon (LV04, LV09, LV10, LV11, LV16, LV22, LV23, LV24, LV25, LV26, LV27, LV29, LV37, LV42, LV43, LV45, LV46, LV49, LV53, LV54, LV55 and LV56); and Group IV—Basically compound by the stations located in the central region of each sector (LV05, LV06, LV07, LV14, LV19, LV20, LV30, LV31, LV44, LV47, LV48, LV50, LV51 and LV52).

## Discussion

### Characteristics of physicochemical water parameters at Vermelha Lagoon

Vermelha Lagoon displayed salinities lower than those reported by [6] and on average similar to Araruama Lagoon [49]. The sampling events occurred during the rainfall period. The rainfall increase of about 16% in 2013 may have contributed to salinities <100 (CILSJ, 2013). In the dry season, subsequently to the sampling events of this study, salinities >120 in the central and western Vermelha Lagoon sectors were recorded. The presence of salt pans and salt extraction laboring may interfere in the salinity of the eastern sector of the lagoon (Fig 1). The salinity distribution map indicates that, lower values were recorded in the eastern sector compared to the other lagoon compartments (Fig 2). This effect may be attributed to water capture for salt pan activities and water exchanges with Araruama Lagoon though a channel (see Fig 1). The effects of salinity decreases are observed in field, with corrosion and bleaching (death) of stromatolites structures. Dissolved oxygen concentrations are a very important factor for the maintenance of aquatic life, and it is one of the main water and sedimentary environmental quality indicators. The dissolved oxygen values registered (sediment/water layer) at Vermelha Lagoon in this work (5.20 mg L^-1^ to 10.34 mg L^-¹^) were higher than in all the other Rio de Janeiro lagoons, such as Maricá Lagoon[50] [19], Araruama Lagoon [49] and Itaipu Lagoon [19]. Low dissolved oxygen values in sedimentary/water layer are commonly found in hypersaline lagoons that display human impacts. Martins et al. [17] reported dissolved oxygen values of 0.16–0.55 mg L^-1^ for Bizerte Lagoon (Tunisia), which is affected by municipal, agricultural and industrial activities. The values recorded at Vermelha Lagoon suggest a favorable environment for the establishment of aerobic organisms belonging to several trophic levels. However, Vasconcelos and McKenzie [51] observed that oxygen concentrations of the water in Vermelha Lagoon are deeply reduced, reaching zero during the night.

Temperatures (23.6–31.5°C) were relatively high throughout the sampling period at Vermelha Lagoon. These high temperatures might be related to the low water depths, about 0.5 m in most marginal stations, of this lagoon. The lowest temperatures were recorded in deeper stations (>2m) in the central areas of each lagoonal compartment (Fig 2).

Alkaline and hypersaline environments like Vermelha Lagoon may be limiting for biota diversity. In this environment, the proliferation of certain kinds of halophilic archaebacteria, particularly *Natronobacteria*, which optimally grows at pH 9.5 [52], causes the salt flats of Vermelha Lagoon to present a pink color, as described by Silva e Silva et al. [9]. *Natronobacteria* can tolerate both halophilic and alkaline environments, meaning that they can thrive in both salty and high pH environments [53]. The most alkaline region of the lagoon was in the shallow area, close to salt pans, where a large surface covered by microbial mats can be observed, and in the stromatolite field. The only exception was at station LV56 (Fig 2).

### Factors influencing the sedimentary organic matter at Vermelha Lagoon

According to the Brazilian CONAMA 357 Resolution [54], the acceptable phosphorus content in Brazilian saline environments is 62 μg g^-1^. At Vermelha Lagoon, the mean TP value of 78.46 ± 40.89 μg g^-1^, much higher than the limit recommended by CONAMA 357 [54]. The highest TP contents were recorded at the western side of each lagoonal compartment and near the central area, close to the salt pans (Fig 2). Lanza-Espino [55] found phosphorus values ranging from 526 μg g^−1^ to 1238.8 μg g^−1^ at Altata Enseñada del Pabelon Lagoon (Mexico). Borges et al. [56] observed values of 1196 ± 355 μg g^-1^ at Guanabara Bay. Even with values above the CONAMA 357 [54] recommendation, Vermelha Lagoon is not so much more impacted than other regions considered polluted. The excessive increase of phosphorous concentrations is probably related to agriculture runoff, as observed by Datta et al. [57] at the Ganges-Brahmaputra-Meghna river basin, in India or to domestic liquid effluents as observed by Martins et al. [16] at Ria de Aveiro (Portugal). High phosphorous concentrations may lead to high primary productivity and, ultimately, to eutrophication, resulting in high accumulation of organic matter on the bottom and dysoxic conditions in the water column. Most of the stations evaluated herein presented relatively high TOC concentrations (1.2–7.10%; mean 3.40%) and values <1% were observed in only two stations (LV13 and LV15). Vermelha Lagoon has moderately to heavy organic enrichment according to Teodoro et al. [58]. Vermelha Lagoon also displays relatively high sedimentary TOC content. For instance, Paez-Osuna et al. [59] found TOC values ranging from 0.28% to 11.15% in areas affected by effluents originated by agriculture and sugar-cane industries at Altata Enseñada del Pabelon Lagoon, on the northwest coast of Mexico. Siqueira et al. [60] documented TOC concentrations ranging from 0.09% to 5.78% at the Santos estuarine system (Brazil), a region comprising a densely urbanized area and the largest Brazilian Industrial Complex (petrochemical, steel, and fertilizer industries), and the main Latin American Harbor (Santos Port). At Bizerte Lagoon (Tunisia), an area affected by municipal, agricultural and industrial activities, Martins et al. [17] reported TOC content ranging from 2.54%– 5.93%. According to Mendonça-Filho et al. [34], sediments with TOC content >2.5% and high organic matter accumulation rates should in general become dysoxic to anoxic environments. A high supply of organic matter and its degradation can lead to depletion of dissolved oxygen [61]. Thus, it is expected that the bottom sediments at Vermelha Lagoon may be dysoxic or anoxic environments and might affect the proliferation and viability of living organisms.

However, the surface sediments of Vermelha Lagoon present relatively low TS contents (0.02% to 0.7%; mean 0.3 ± 0.13%) when compared to other Brazilian lagoonal systems, such as Itaipu Lagoon (0.03–1.73%; mean 1.00 ± 0.59% [19]), Guanabara Bay (mean 1.4% [20]) and Santos Estuary (mean 6.03% [60]). Martins et al. [17] also documented low TS values (0.04%) at Bizerte Lagoon, located in a semi-arid climatic zone of the Mediterranean coast of Tunisia.

The TOC/S ratio values (8.96–20) are relatively high at Vermelha Lagoon. A TOC/S ratio >3 indicates oxidizing environments and <3 indicates reducing conditions [62]. The predominance of reducing processes depends on the amount of organic matter and sulfur availability [63], [64]. Thus, the TOC/S values observed herein are indicative that the surface sediments of Vermelha Lagoon present oxidizing conditions, despite containing high sedimentary TOC content. Oxidizing conditions were also identified at Bizerte Lagoon by Martins et al. [17], where the TOC/S ratio reached values up to 24. The oxygen on surface sediment can be provided by the high primary production of cyanobacteria and diatoms that also contribute to water column oxygenation during day.

### Factors controlling the distribution of biopolymers at Vermelha Lagoon

BPC concentrations (CHO + PTN +LIP) in marine environments is a tool used for the biochemical characterization and interpretation of organic matter origin accumulated on the sediment [65]. This variable is also useful to access the quality and quantity of food availability for benthic organisms [66]. The BPC range (15.01–43.36 mg C g^-1^) at Vermelha Lagoon is high when compared to other hypersaline ecosystems, such as Bizerte Lagoon [17]. High BPC values and large local variations are expected in environments with high primary biological productivity [67]. The highest BPC content recorded at Vermelha Lagoon are equivalent to aquatic environments with large sewage discharges, such as Guanabara Bay [68], [69], [70], [18], [21], [20], the Paraiba do Sul River Delta [71] and Itaipu Lagoon [19].

The composition of organic matter in biopolymers in coastal areas is commonly characterized by small amounts of LIP and large quantities of PTN, frequently exceeding CHO concentrations [72]. Vermelha Lagoon presents however higher average concentrations of biopolymers (CHO mean 16.81 ± 7.12 mg C g^-1^; LIP mean 1.86 ± 9.72 mg C g^-1^ and; PTN mean 1.13 ± 4.79 mg C g^-1^) than other coastal regions worldwide. Danovaro et al. [66] revealed CHO concentrations ranging from 6.2–575.8 mg C g^-1^, PTN from 0.70–33.6 mg C g^-1^ and LIP from 0.26–3.75 mg C g^-1^ in sediments of the Aegean Sea. This area receives the nutrient-rich surface waters of the Black Sea, but the southern area is probably the most oligotrophic part of the entire Mediterranean Sea [73]. By studying two nearby estuarine systems located in the inner zone of Bay of Biscay (N Spain), Cotano and Villate [74] reported values in Mundaka Estuary and Bilbao estuary for: CHO ranging from 0.02–0.57 mg C g^-1^ and 0.05–1.20 mg C g^-1^, respectively; PTN contents of 0.00–1.67 mg C g^-1^ and 0.06–5.61 mg C g^-1^, respectively and; LIP contents between 0.03–0.50 mg C g^-1^ and 0.97–2.54 mg C g^-1^, respectively. The Mundaka Estuary preserves valuable estuarine habitats by supporting a rich wildlife. In this estuary, urban and industrial developments are sources of pollution, but the presence of a sewage treatment plant in the upper estuary, reduces this negative impact on the environment. On the other hand, the Bilbao region comprises an urbanized area and is affected by navigation commerce and harbor activities, as well as by urban and industrial development. These activities have led to loss of the intertidal zone that was converted into a narrow dredge channel [74]. The excessive increase of PTN and LIP in coastal areas may be associated with anthropogenic organic matter, while CHO is related to phytoplankton and/or has a detrital origin from continental vegeTable sources [74]. The high LIP content in sediments has been also linked with the refractory fraction of organic matter [75], [76], [77], [78]. In lagoons suffering anthropic impacts, the increase of LIP concentrations may be associated with the increment of recalcitrant substances originated by fluvial inputs [19]. Vermelha Lagoon displays relatively high LIP concentrations in the central zone of the lagoonal compartments, which should be related to low hydrodynamics and high anaerobic bacterial activity [19] in these areas. No fluvial inputs are present in the lagoon and the sewage launch is not significant in this region, but it was possible to identify in the field that the fresh water channel sometimes breaks in the western sector.

PTN decomposition is faster than CHO, and, therefore, only new material, recently deposited, presents high PTN values [43], [19]. The high PTN values found in the east margin suggest sewage input from townhouses in the Praia Seca District like was observed in Itaipu lagoon by Laut et al. [19]. Thus, high PTN/CHO ratios should be related to high quality organic matter that might favor benthic consumers [79]. The PTN/CHO ratio is directly linked to the importance of the nitrogen fraction in organic matter [13]. Productive areas, such as estuaries and coastal regions are prone to high PTN/CHO values [74], [20], [19]. PTN/CHO<1 indicate the predominance of aged organic matter and >1 the presence of fresh material of recent origin [80]. Vermelha Lagoon exhibited PTN/CHO<1, indicating that the fresh produced organic matter is being consumed and/or rapidly decomposed.

According to Pusceddu et al. [15] and Dell’Anno [12], the relationship between PTN and CHO can be used also as an indicator of the trophic state of coastal systems: meso-oligotrophic (PTN <1.5 mg C g^-1^; CHO <5 mg C g^-1^), eutrophic (PTN <1.5–4 mg C g^-1^; CHO <5–7 mg C g^-1^) and hyper-trophic (PTN >4 mg C g^-1^; CHO >7 mg C g^-1^). Accordingly, the study area can be classified, in general as eutrophic.

The CHO/TOC ratio is used for the qualitative or semi qualitative distinction of the organic material origin supplied by allochthonous (such as agricultural and domestic effluents) or autochthonous (biological productivity) sources [65]. According to Paez-Osuna et al. [59], in coastal systems, values of CHO/TOC<20 are indicative of organic matter of natural origin, while higher values may be linked with sewage effluents inputs. At Vermelha Lagoon, the values of CHO/TOC<20 suggest that sediments are mostly enriched in organic matter of autochthonous origin, essentially produced by the microbial activity broadly spread through this ecosystem.

### Bottom environment

Based on the Cluster Analysis, it was possible to identify four main regions in Vermelha Lagoon that reflect different environmental conditions (Fig 4): Region I (Group I) composed by stations located in a shallow area of the western side of the lagoon is characterized by high values of salinity (mean 60), TP (mean 145 μg g^-1^) and LIP (mean 6.2 mg C g^-1^) (S2 Table). Sediments of this region are enriched in recalcitrant substances which should be introduced by the fresh water channel breakdown. The means of TP and LIP indicate high anaerobic activity in this region [19]. The Region II (Group II) represents the influence of salt pans where the highest values of BPC (mean 33.7 mg C g^-1^), specially CHO (mean 27.2 mg C g^-1^), were found (S2 Table). This region represent of Vermelha Lagoon with greater activity of halophilic archaebacteria and cyanobacteria as observed by Silva e Silva et al. [9]. In this region the autotrotrophic activity should be higher than the heterotrophic processes. The Region III (Group III) with stations spread for all the lagoon is characterized by high values of dissolved oxygen (mean 7.1 mg L^-1^) and lower values of TP (mean 42.8 μg g^-1^) (S2 Table). At some marginal stations (LV26, LV27 and LV29) of this sector the growth of domal and biscuit stromatolites and extensive microbial mattes where observed. Region IV (Group IV) where the highest values of TOC (mean 4%) and TS (mean 0.3%) were found are depocenters of organic matter accumulated in general in deeper zones of the lagoon (S2 Table). These characteristics can also be seen in the eastern sector highlighted the area influenced by the Araruama communication channel in Vermelha Lagoon (Fig 4B).

## Conclusions

Vermelha Lagoon is an alkaline environment, with good oxygenation conditions of water column and surface sediment. This lagoon can be considered an eutrophic ecosystem with high concentration of autochthonous organic matter. The relationships established between the biopolymer components indicated that the autotrophic activity is more intense than heterotrophic activity, and this organic matter produced is not degraded because the hypersaline conditions. The bottom sediments of central sectors concentrate high values of organic matter (namely CHO and LIP) and sulphur. The impacted area regarding salt pan activity was highlighted by high BPC concentrations, mainly CHO. The recorded decrease in salinity found in the sector of stromatolites and microbial mattes field of the lagoon poses a threat to the survival of these organisms which contribute to high oxygen concentrations and autotrophic production in the lagoon.

## Supporting information

S1 Table Values of dissolved oxygen (O), temperature (T), pH, salinity (Sal) and measured in each site are also reported. The sedimentary content in total organic carbon (TOC), total sulphur (TS), carbohydrates (CHO), lipids (LIP) and proteins (PTN), total biopolymeric carbon (BPC), percentage of biopolymeric carbon (BPC) and total phosphorus (TP) are presented. This table also shows the ratio values: PTN/CHO, TOC/TS and CHO/TOC.
(DOCX)

S2 Table Maximum, mean and standard derivation values oxygen (O), temperature (T), pH, salinity (Sal), total organic carbon (TOC), total sulfur (TS), carbohydrates (CHO), lipid (LIP), protein (PTN), total biopolymeric carbon (BPC), total phosphorus (TS) and the ration values of PTN/CHO, TOC/TS and CHO/TOC in each group of stations identified by cluster analysis included in Fig 4. (DOCX)
