# Supplementary material for: Assessment of the trophic state of a hypersaline-carbonatic environment: Vermelha Lagoon (Brazil)
Source: PLoS One. 2017 Sep 21;12(9):e0184819. doi: 10.1371/journal.pone.0184819 (PMC5608279; doi:10.1371/journal.pone.0184819)
Supplement: S1 Table — The sedimentary content in total organic carbon (TOC), total sulphur (TS), carbohydrates (CHO), lipids (LIP) and proteins (PTN), total biopolymeric carbon (BPC), percentage of biopolymeric carbon (BPC) and total phosphorus (TP) are presented. This table also shows the ratio values: PTN/CHO, TOC/TS and CHO/TOC. (DOCX) [file pone.0184819.s001.docx]

| **Stations** | **Lat** | **Long** | **O** | **T** | **pH** | **Sal** | **TOC** | **TS** | **CHO** | **LIP** | **PTN** | **BPC** | **TP** | **PTN/CHO** | **TOC/TS** | **CHO/TOC** |
| --- | --- | --- | --- | --- | --- | --- | --- | --- | --- | --- | --- | --- | --- | --- | --- | --- |
|  |  |  | mg/L | ºC |  |  | % | % | mg C g^-1^ | mg C g^-1^ | mg C g^-1^ | mg C g^-1^ | µg/g |  |  |  |
| LV01 | 22.93193791 | 42.40388236 | 7.1 | 25.7 | 8.1 | 56.68 | 1.79 | 0.15 | 2.5 | 3.79 | 1.81 | 8.1 | 151.96 | 0.3 | 12.29 | 1.81 |
| LV02 | 22.93047361 | 42.40437746 | 6.9 | 25.4 | 8.2 | 58.79 | 2.3 | 0.24 | 2.84 | 4.79 | 1.21 | 8.84 | 128.76 | 0.35 | 9.6 | 1.54 |
| LV03 | 22.93154089 | 42.40053027 | 6.5 | 25.8 | 8.1 | 58.9 | 1.47 | 0.13 | 2.73 | 5.08 | 1.05 | 8.86 | 182.08 | 0.31 | 11.31 | 2.41 |
| LV04 | 22.93024937 | 42.40068256 | 7.3 | 25.2 | 8.1 | 59.19 | 4.42 | 0.48 | 4.56 | 6.39 | 1.32 | 12.27 | 47.07 | 0.24 | 9.21 | 1.11 |
| LV05 | 22.92885935 | 42.40071764 | 7.1 | 24.9 | 8.2 | 61.97 | 1.93 | 0.15 | 3.23 | 4.25 | 1.44 | 8.92 | 88.4 | 0.36 | 12.87 | 1.85 |
| LV06 | 22.9286983 | 42.39713793 | 6.8 | 25.2 | 8 | 62.37 | 2.9 | 0.29 | 3.05 | 6.85 | 0.96 | 10.86 | 79.43 | 0.26 | 10 | 1.5 |
| LV07 | 22.93006962 | 42.39726831 | 5.8 | 24.1 | 8.1 | 60.55 | 4.14 | 0.41 | 3.6 | 6.05 | 1.33 | 10.98 | 70.36 | 0.3 | 10.1 | 1.06 |
| LV08 | 22.93133043 | 42.39705772 | 7.4 | 25 | 8.09 | 59.97 | 4.68 | 0.44 | 5.63 | 6.15 | 1.26 | 13.04 | 125.74 | 0.18 | 10.64 | 1.11 |
| LV09 | 22.93232557 | 42.39703151 | 7.9 | 25 | 8.1 | 59.82 | 2.44 | 0.23 | 3.68 | 4.35 | 1.07 | 9.1 | 39.46 | 0.24 | 10.61 | 1.49 |
| LV10 | 22.9322017 | 42.39385353 | 8.6 | 24.7 | 8.1 | 60.16 | 2.27 | 0.18 | 4.47 | 4.08 | 1.78 | 10.33 | 47.46 | 0.33 | 12.61 | 1.82 |
| LV11 | 22.93114216 | 42.39395955 | 6 | 24.3 | 8.1 | 60.68 | 2.15 | 0.24 | 5.46 | 6.65 | 1.52 | 13.63 | 58.57 | 0.23 | 8.96 | 2.53 |
| LV12 | 22.92998069 | 42.39398267 | 6.2 | 24.1 | 8.1 | 60.55 | 4.01 | 0.44 | 4.4 | 6.63 | 1.67 | 12.7 | 142.99 | 0.31 | 9.11 | 1.27 |
| LV13 | 22.93283682 | 42.39291356 | 6.4 | 25.6 | 8.1 | 60.42 | 0.28 | 0.02 | 5.5 | 0.75 | 0.75 | 7 | 137.73 | 0.11 | 14 | 10 |
| LV14 | 22.93059525 | 42.39353006 | 7.2 | 25 | 7.9 | 62.64 | 2.48 | 0.21 | 4.69 | 5.21 | 1.37 | 11.27 | 75.92 | 0.24 | 11.81 | 1.82 |
| LV15 | 22.9279024 | 42.39362437 | 6.8 | 25 | 8 | 63.1 | 0.99 | 0.07 | 2.88 | 2.48 | 1.18 | 6.54 | 148.74 | 0.33 | 14.14 | 2.64 |
| LV16 | 22.92792056 | 42.38948891 | 7.2 | 24.6 | 8.1 | 61.71 | 2.67 | 0.27 | 3.26 | 4.63 | 1.21 | 9.1 | 34.39 | 0.3 | 9.89 | 1.36 |
| LV17 | 22.93042827 | 42.38979751 | 7.6 | 25 | 8 | 61.42 | 4.03 | 0.4 | 3.59 | 5.77 | 0.87 | 10.23 | 139.68 | 0.2 | 10.08 | 1.17 |
| LV18 | 22.93264735 | 42.38980084 | 6.5 | 25 | 8.1 | 61.42 | 3.53 | 0.34 | 5.09 | 6.91 | 0.87 | 12.87 | 50.18 | 0.14 | 10.38 | 1.46 |
| LV19 | 22.93245789 | 42.38668869 | 6.8 | 25 | 8.1 | 61.95 | 4.38 | 0.39 | 6.1 | 6.33 | 1 | 13.43 | 65 | 0.13 | 11.23 | 1.23 |
| LV20 | 22.93014152 | 42.38656205 | 7 | 24 | 8.1 | 62.28 | 6.88 | 0.7 | 5.64 | 6.73 | 1.02 | 13.39 | 81.57 | 0.15 | 9.83 | 0.78 |
| LV21 | 22.92761596 | 42.3869382 | 6.9 | 25 | 8.1 | 61.03 | 3.62 | 0.32 | 10.97 | 5.79 | 0.85 | 17.61 | 150.5 | 0.06 | 11.31 | 1.94 |
| LV22 | 22.92727714 | 42.38334874 | 6.9 | 24.8 | 8 | 62.29 | 3.62 | 0.32 | 7.4 | 6.64 | 0.74 | 14.78 | 36.05 | 0.08 | 11.31 | 1.63 |
| LV23 | 22.92998596 | 42.38351256 | 7 | 24.8 | 8.1 | 61.91 | 3.36 | 0.31 | 8.18 | 2.59 | 0.96 | 11.73 | 53.69 | 0.1 | 10.84 | 1.4 |
| LV24 | 22.93222895 | 42.3834169 | 6.8 | 25 | 8.1 | 61.19 | 4.2 | 0.4 | 7.09 | 2.53 | 0.88 | 10.5 | 25.13 | 0.1 | 10.5 | 1 |
| LV25 | 22.93180645 | 42.38041983 | 6.4 | 25 | 8.1 | 62.64 | 3.83 | 0.37 | 6.68 | 1.53 | 0.72 | 8.93 | 23.57 | 0.09 | 10.35 | 0.93 |
| LV26 | 22.92970105 | 42.38030955 | 6.8 | 25.2 | 8 | 62.37 | 4.11 | 0.4 | 5.69 | 1.67 | 0.7 | 8.06 | 23.38 | 0.1 | 10.28 | 0.78 |
| LV27 | 22.92817813 | 42.3782701 | 6.9 | 25.2 | 8.1 | 62.68 | 4.27 | 0.46 | 5.67 | 1.97 | 1.13 | 8.77 | 43.75 | 0.16 | 9.28 | 0.82 |
| LV28 | 22.92964263 | 42.37787257 | 7.1 | 24.9 | 8.2 | 61.97 | 2.92 | 0.22 | 15.35 | 1.84 | 0.69 | 17.88 | 79.04 | 0.13 | 13.27 | 2.45 |
| LV29 | 22.93164656 | 42.37779465 | 6.8 | 26 | 8.1 | 61.03 | 2.13 | 0.2 | 5.53 | 1.72 | 0.66 | 7.91 | 55.06 | 0.1 | 10.65 | 1.49 |
| LV30 | 22.93065509 | 42.37773691 | 7.8 | 30 | 9.2 | 51 | 6 | 0.3 | 7.63 | 1.78 | 0.85 | 10.26 | 67.05 | 0.1 | 20 | 0.67 |
| LV31 | 22.93057684 | 42.37632132 | 6.6 | 25.5 | 7.9 | 54.57 | 5.6 | 0.42 | 9.73 | 1.72 | 1.04 | 12.49 | 58.67 | 0.09 | 13.33 | 0.92 |
| LV32 | 22.92893748 | 42.3771972 | 6.9 | 24 | 8 | 54.75 | 5.62 | 0.44 | 8.23 | 4.72 | 1.13 | 14.08 | 45.6 | 0.11 | 12.77 | 1 |
| LV33 | 22.92919257 | 42.37600793 | 5.2 | 31.5 | 8.4 | 55 | 3.7 | 0.29 | 8.3 | 3.43 | 1.08 | 12.81 | 126.03 | 0.1 | 12.76 | 1.38 |
| LV34 | 22.93200859 | 42.37554822 | 7.4 | 25.8 | 7.9 | 55.06 | 1.81 | 0.14 | 8.43 | 2.15 | 1.03 | 11.61 | 126.52 | 0.1 | 12.93 | 2.57 |
| LV35 | 22.9302038 | 42.37504557 | 7.9 | 25 | 8.8 | 56.88 | 5.52 | 0.37 | 13 | 4.81 | 2.19 | 20 | 124.27 | 0.14 | 14.92 | 1.45 |
| LV36 | 22.9311276 | 42.37443766 | 5.8 | 24.6 | 7.9 | 53.76 | 4.25 | 0.29 | 10.49 | 3.39 | 2.04 | 15.92 | 89.08 | 0.16 | 14.66 | 1.5 |
| LV37 | 22.93129334 | 42.37288966 | 6.7 | 23.8 | 7.7 | 54.37 | 7.1 | 0.48 | 5.98 | 2.72 | 2.22 | 10.92 | 16.07 | 0.29 | 14.79 | 0.61 |
| LV38 | 22.93145855 | 42.37182024 | 8.6 | 24 | 8.1 | 53.24 | 2.68 | 0.24 | 7.26 | 1.42 | 1.29 | 9.97 | 123.49 | 0.15 | 11.17 | 1.49 |
| LV39 | 22.93026146 | 42.37114672 | 7.3 | 25.6 | 8 | 43.37 | 1.45 | 0.12 | 11.57 | 2.4 | 1.55 | 15.52 | 124.37 | 0.11 | 12.08 | 4.28 |
| LV40 | 22.92849793 | 42.37086229 | 7.8 | 24 | 8.2 | 53.24 | 2.74 | 0.22 | 11.06 | 2.41 | 1.23 | 14.7 | 105.26 | 0.09 | 12.45 | 2.15 |
| LV41 | 22.92626938 | 42.36965726 | 6.3 | 23.6 | 8.2 | 53.77 | 3.61 | 0.38 | 12.22 | 1.45 | 1.15 | 14.82 | 102.63 | 0.08 | 9.5 | 1.64 |
| LV42 | 22.92782448 | 42.36926196 | 7.2 | 26 | 8.3 | 53.02 | 1.2 | 0.08 | 11.06 | 2.02 | 1.05 | 14.13 | 34.39 | 0.08 | 15 | 4.71 |
| LV43 | 22.92947253 | 42.36958205 | 6.4 | 24 | 8.1 | 53.01 | 2.84 | 0.28 | 6.35 | 3.13 | 0.64 | 10.12 | 41.61 | 0.08 | 10.14 | 1.43 |
| LV44 | 22.93112133 | 42.36991626 | 8 | 24.9 | 8.2 | 52.43 | 3.05 | 0.27 | 7.61 | 2.93 | 0.55 | 11.09 | 88.3 | 0.06 | 11.3 | 1.45 |
| LV45 | 22.93256827 | 42.37039007 | 7.1 | 24.7 | 8.1 | 45.33 | 3.55 | 0.27 | 8.02 | 2.29 | 0.87 | 11.18 | 43.26 | 0.09 | 13.15 | 1.26 |
| LV46 | 22.9335077 | 42.36730894 | 6.4 | 26.4 | 7.9 | 52.67 | 3.22 | 0.3 | 5.97 | 2.86 | 0.6 | 9.43 | 44.04 | 0.08 | 10.73 | 1.17 |
| LV47 | 22.93169828 | 42.36748412 | 6.1 | 26 | 8 | 52.59 | 3.31 | 0.27 | 7.21 | 4.33 | 0.77 | 12.31 | 68.32 | 0.09 | 12.26 | 1.49 |
| LV48 | 22.92988656 | 42.3676194 | 6.2 | 25.6 | 8.2 | 52.44 | 7.06 | 0.53 | 8.85 | 3.68 | 0.96 | 13.49 | 66.95 | 0.09 | 13.32 | 0.76 |
| LV49 | 22.92796279 | 42.3677219 | 6.4 | 25.2 | 7.8 | 52.66 | 2.83 | 0.3 | 5.35 | 1.99 | 0.6 | 7.94 | 61.98 | 0.09 | 9.43 | 1.12 |
| LV50 | 22.92622214 | 42.36775288 | 6.2 | 25 | 8 | 52.36 | 2.27 | 0.19 | 5.24 | 1.39 | 0.73 | 7.36 | 90.64 | 0.11 | 11.95 | 1.3 |
| LV51 | 22.92598551 | 42.36573335 | 6.7 | 24.8 | 8.2 | 45.63 | 2.35 | 0.19 | 5.7 | 3.23 | 1.43 | 10.36 | 72.61 | 0.2 | 12.37 | 1.76 |
| LV52 | 22.92779728 | 42.36561772 | 6.8 | 24 | 8.2 | 52.94 | 4.78 | 0.35 | 9.61 | 5.13 | 1.65 | 16.39 | 65.39 | 0.14 | 13.66 | 1.37 |
| LV53 | 22.92957131 | 42.36546488 | 6 | 24.9 | 7.94 | 50.09 | 6.02 | 0.46 | 7.62 | 1.48 | 1.58 | 10.68 | 43.85 | 0.17 | 13.09 | 0.71 |
| LV54 | 22.93136471 | 42.36533075 | 6.5 | 25.5 | 8.2 | 52.44 | 1.84 | 0.13 | 6.01 | 1.84 | 1.75 | 9.6 | 45.31 | 0.24 | 14.15 | 2.09 |
| LV55 | 22.93313714 | 42.36515837 | 9.2 | 26.8 | 8.6 | 52.6 | 1.57 | 0.11 | 4.98 | 2.34 | 2.08 | 9.4 | 44.58 | 0.34 | 14.27 | 2.39 |
| LV56 | 22.93217259 | 42.3632906 | 10.36 | 24.3 | 8.2 | 50.34 | 1.9 | 0.12 | 7.58 | 2.56 | 2.34 | 12.48 | 46.4 | 0.25 | 15.83 | 1.99 |
