# Supplementary material for: Assessment of the trophic state of a hypersaline-carbonatic environment: Vermelha Lagoon (Brazil)
Source: PLoS One. 2017 Sep 21;12(9):e0184819. doi: 10.1371/journal.pone.0184819 (PMC5608279; doi:10.1371/journal.pone.0184819)
Supplement: S2 Table — (DOCX) [file pone.0184819.s002.docx]

|  |  | **O** | **T** | **pH** | **Sal** | **TOC** | **TS** | **CHO** | **LIP** | **PTN** | **BPC** | **TP** | **PTN/CHO** | **TOC/TS** | **CHO/TOC** |
| --- | --- | --- | --- | --- | --- | --- | --- | --- | --- | --- | --- | --- | --- | --- | --- |
| REGION I | Means | 6.9 | 25.2 | 8.1 | 60.0 | 2.4 | 0.2 | 9.4 | 6.2 | 2.5 | 18.1 | 144.7 | 0.3 | 11.4 | 2.7 |
|  | Standard deviation | 0.5 | 0.6 | 0.1 | 1.9 | 1.6 | 0.2 | 3.2 | 2.9 | 0.7 | 4.3 | 17.6 | 0.1 | 1.9 | 3.0 |
|  | Maximum value | 7.6 | 25.8 | 8.2 | 63.1 | 4.7 | 0.4 | 14.1 | 9.7 | 3.7 | 24.9 | 182.1 | 0.4 | 14.1 | 10.0 |
|  | Minimum value | 6.2 | 24.1 | 8.0 | 56.7 | 0.3 | 0.0 | 6.3 | 1.0 | 1.5 | 12.9 | 125.7 | 0.1 | 9.1 | 1.1 |
|  |  |  |  |  |  |  |  |  |  |  |  |  |  |  |  |
| REGION II | Means | 7.0 | 25.4 | 8.2 | 54.7 | 3.2 | 0.3 | 27.2 | 3.9 | 2.7 | 33.7 | 115.1 | 0.1 | 12.5 | 2.1 |
|  | Standard deviation | 1.0 | 2.3 | 0.3 | 5.1 | 1.2 | 0.1 | 6.0 | 1.9 | 1.0 | 6.7 | 21.0 | 0.0 | 1.6 | 0.9 |
|  | Maximum value | 8.6 | 31.5 | 8.8 | 62.0 | 5.5 | 0.4 | 38.4 | 7.7 | 4.5 | 43.4 | 150.5 | 0.2 | 14.9 | 4.3 |
|  | Minimum value | 5.2 | 23.6 | 7.9 | 43.4 | 1.5 | 0.1 | 18.1 | 1.9 | 1.4 | 22.7 | 79.0 | 0.1 | 9.5 | 1.4 |
|  |  |  |  |  |  |  |  |  |  |  |  |  |  |  |  |
| REGION II | Means | 7.1 | 25.0 | 8.1 | 57.2 | 3.4 | 0.3 | 15.6 | 4.6 | 2.4 | 22.6 | 42.8 | 0.2 | 11.6 | 1.5 |
|  | Standard deviation | 1.0 | 0.7 | 0.2 | 5.1 | 1.4 | 0.1 | 4.2 | 2.5 | 1.0 | 4.2 | 12.0 | 0.1 | 2.0 | 0.8 |
|  | Maximum value | 10.4 | 26.8 | 8.6 | 62.7 | 7.1 | 0.5 | 27.6 | 9.2 | 4.8 | 32.5 | 65.0 | 0.3 | 15.8 | 4.7 |
|  | Minimum value | 6.0 | 23.8 | 7.7 | 45.3 | 1.2 | 0.1 | 8.1 | 2.0 | 1.2 | 16.8 | 16.1 | 0.1 | 9.0 | 0.6 |
|  |  |  |  |  |  |  |  |  |  |  |  |  |  |  |  |
| REGION IV | Means | 6.8 | 25.3 | 8.2 | 55.7 | 4.1 | 0.3 | 15.7 | 5.5 | 2.2 | 23.4 | 74.9 | 0.2 | 12.5 | 1.3 |
|  | Standard deviation | 0.6 | 1.5 | 0.3 | 5.6 | 1.8 | 0.2 | 5.8 | 2.4 | 0.7 | 5.2 | 10.1 | 0.1 | 2.6 | 0.4 |
|  | Maximum value | 8.0 | 30.0 | 9.2 | 62.6 | 7.1 | 0.7 | 24.3 | 9.1 | 3.4 | 34.2 | 90.6 | 0.4 | 20.0 | 1.9 |
|  | Minimum value | 5.8 | 24.0 | 7.9 | 45.6 | 1.9 | 0.2 | 7.6 | 1.9 | 1.1 | 16.5 | 58.7 | 0.1 | 9.8 | 0.7 |
|  |  |  |  |  |  |  |  |  |  |  |  |  |  |  |  |
